# CXCR6^+^ and NKG2C^+^ Natural Killer Cells Are Distinct With Unique Phenotypic and Functional Attributes Following Bone Marrow Transplantation

**DOI:** 10.3389/fimmu.2022.886835

**Published:** 2022-06-29

**Authors:** Kevin Aviles-Padilla, Laura S. Angelo, Dwight Fan, Silke Paust

**Affiliations:** ^1^ Center for Human Immunobiology, Department of Pediatrics, Texas Children’s Hospital, Houston, TX, United States; ^2^ The Developing Investigative Scholar’s Program (DISP), Center for Human Immunobiology, Department of Pediatrics, Texas Children’s Hospital and Rice University, Houston, TX, United States; ^3^ Department of Immunology and Microbiology, The Scripps Research Institute, La Jolla, CA, United States

**Keywords:** natural killer cell, human cytomegalovirus, CXCR6, NKG2C, bone marrow transplantation, hematopoietic stem cell transplantation

## Abstract

Reactivation of human cytomegalovirus (HCMV) is a life-threatening complication in transplant patients. Natural Killer (NK) cells are the first lymphocyte lineage to reconstitute following an allogeneic hematopoietic stem cell transplant (HSCT). Amongst them, NK cell Group 2 isoform C/Killer cell lectin-like receptor subfamily C, member 2 (NKG2C)-expressing NK cells contribute significantly to patient protection upon HCMV reactivation. NKG2C^+^ NK cells are capable of immunological memory, albeit NK cell memory is not restricted to them. Hepatic C-X-C Motif Chemokine Receptor 6 (CXCR6)-expressing NK cells also mediate memory responses in mice and humans. Small numbers of them circulate and can thus be studied in peripheral blood samples. We hypothesize that NKG2C^+^ and CXCR6^+^ NK cell subsets are distinct. To test our hypothesis, we used multi-parametric flow cytometry to determine the phenotypes and effector functions of CD56^bright^ vs. CD56^dim^ and NKG2C^+^ vs. CXCR6^+^ human NK cell subsets in the peripheral blood (PB) of pediatric transplant recipients monthly while monitoring patients for HCMV reactivation. Interestingly, we did not find any NKG2C^+^CXCR6^+^ NK cells in the transplant recipients’ peripheral blood, suggesting that NKG2C^+^ and CXCR6^+^ NK cells are distinct. Also, NKG2C–CXCR6– NK cells, rather than NKG2C^+^ NK cells, made up most NK cells post-transplant, even in transplant recipients with HCMV viremia. In contrast to NKG2C^+^ NK cells, CXCR6^+^ NK cells appeared phenotypically less differentiated but were highly proliferative and produced IFN-γ and TNF*_α_*. Our findings contribute to our understanding of post-transplant NK cell development and its implications for human health.

## Introduction

NK cells are an essential cytotoxic immune cell type responsible for host protection against viral infection and malignancies (1–6). NK cells develop from bone marrow-derived hematopoietic stem cells and arise from a common lymphoid progenitor but do not express T or B cell receptors (3, 6, 7). Instead, NK cells express a diverse receptor repertoire, including inhibitory and activating receptors. NK cell-expressed inhibitory receptors include NKG2A/Cluster of Differentiation (CD) 94, which recognizes the non-classical human leukocyte antigen (HLA)-E molecule, and inhibitory Killer Ig-like Receptors (KIRs) that recognize HLA-A, B, and C cell surface molecules (8). These interactions license NK cells during their development for functional competence (9, 10), protect healthy cells from NK-mediated lysis (11) and allow NK cells to recognize a reduction in HLA Class 1 molecule expression resulting from infection or malignant transformation (12). NK cells also express a variety of activating receptors, including lectin-like receptors NKp80, receptors of the NKG2 family, Fc receptors such as CD16 (FcγRIII), the mediator of antibody-dependent cellular cytotoxicity (ADCC), and natural cytotoxicity receptors ((NCRs), including NKp30, NKp44, NKp46 that allow NK cells to recognize an increased expression of stress ligands, leading to NK cell-mediated killing (11, 13). NKG2C, like NKG2A, forms a heterodimer with CD94 on the surface of human NK cells. However, the NKG2C/CD94 complex acts as an activating receptor recognizing the nonclassical Human Leukocyte Antigen-E (HLA-E), which presents signal peptides derived from other Class I HLA proteins (14).

In humans, NK cells are defined as CD56^+^CD3^–^ cells that do not express monocyte (CD14^–^) or B cell lineage markers (CD19^–^). Specific NK cell receptor combinations classify NK cells into distinct subsets that differ in their phenotypes, functions, and/or tissue localizations (15–24). During their development, NK cells progressively acquire the expression of NCAM (CD56), CD16, the activating receptor NKG2D, and the TNF family member and costimulatory CD27 (25). They also start expressing CD62L, a selectin essential for NK cell migration to lymph nodes (26). Less differentiated NK cells (CD56^bright^/CD16^dim^/NKG2A^+^) develop into tissue-specific subpopulations (27). More differentiated NK cells (CD56^dim^/CD16^bright^) in these tissues and peripheral blood (PB) (28, 29) express killer cell immunoglobulin receptors (KIRs), secrete proinflammatory cytokines, perforins, granzymes, defensins, and cathelicidin, and mediate ADCC (30). CD56^dim^ NK cells account for the majority (85-95%) of the circulating NK cell population, while CD56^bright^ NK cells are abundant in secondary lymphoid and peripheral tissues. Cytokine signaling can differentiate and/or activate NK cells, and tissue-specific chemokine receptors recruit NK cells to infected tissues (11).

Upon their activation, CD56^bright^ NK cells are robust cytokine producers and secrete large amounts of IFN-γ and TNF-α (6, 19, 23). In contrast, CD56^dim^ NK cells express high levels of a pore-forming molecule called perforin and granzymes, enzymes that induce apoptosis in target cells. As such, CD56^dim^ NK cells are potent cytotoxic effector cells (31). NK cells express the T-box transcription factors T-bet and Eomesodermin (Eomes), which play an essential role in NK cell development and lineage determination (32). Tissue-resident human NK cells are generally CD56^high^, CXCR6^+^, NKG2D^+^, CD69^+^, and CD16^low^, and express significant levels of Eomes and varied T-bet expression (32, 33). In contrast, circulating human NK cells are generally T-bet^hi^ but Eomes^lo^ (23, 33–36), and express CD49e (20). CD69 is constitutively expressed on most tissue-resident NK cells but can be upregulated upon NK cell activation in any organ (17, 18, 21, 23, 24).

Hematologic malignancies, immune deficiencies, acquired or inherited bone marrow failure syndromes, and hemoglobinopathies are challenging to treat diseases that can be cured with a Hematopoietic Cell Transplant (HCT) or Bone Marrow Transplant (BMT) (37, 38). NK cells are the first lymphocyte to recover after HCT, including in children (39). NK cells are an essential component of post-transplant immunity (40, 41). Their reconstitution efficiency and effector functions affect incidences of common post-transplant complications, including graft-versus-host disease (GVHD), viral infections, and/or cancer relapse (40, 41).

A significant complication after transplant is the reactivation of HCMV, a herpesvirus infection that is generally asymptomatic in healthy people. However, HCMV can become a life-threatening complication in immunocompromised patients and a significant cause of morbidity and mortality (42, 43). Interestingly, NK cell recognition of an HCMV encoded UL-40 protein-derived peptide/HLA-E complex has recently been shown to elicit the expansion of NKG2C^+^ NK cells (44), suggesting that pathogen-derived peptides may modulate NK cell activation through HLA/E-NKG2C interactions. Primary HCMV infection and HCMV reactivation result in the substantial expansion of NKG2C^+^ expressing NK cells that contribute significantly to patient protection (45–52). NKG2C^+^ NK cell frequency directly correlates with HCMV reactivation and outcome (morbidity and mortality risk) in post-transplant patients (45, 46). Generally, expanding NKG2C^+^ NK cells have downregulated their expression of the inhibitory receptor CD94/NKG2A and display a terminally differentiated phenotype (CD57^+^). These cells also frequently express the Killer Immunoglobulin-like Receptor KIR2DL2/DL3 and the costimulator DNAX Accessory Molecule-1 (DNAM-1) and produce significant amounts of IFN-γ (47, 53, 54). It is noteworthy that both maternal and paternal copies of the NKG2C-gene are deleted in about 5% of the human population, and about 20% of people are heterozygous for NKG2C. Both genotypes have been linked to a higher risk of CMV reactivation post-transplant (45, 46, 50, 52).

NK cell-mediated immune memory to CMV has been demonstrated in both mice and humans (47–49, 55, 56). In lymphopenic C57Bl/6 mice, CMV-specific NK cell activation is mediated *via* the NK cell-expressed activating receptor Ly49H, the ligand of which is the murine CMV (mCMV)-encoded protein m157 (57–59), similar to NKG2C^+^ NK cells that expand upon HCMV infection or reactivation in humans. However, adaptive immunity is not restricted to NKG2C/Ly49H^+^ NK cells. We have previously demonstrated that hepatic CXCR6-expressing NK cells mediate adaptive immune responses to viral non-CMV antigens in mice (60–63) and humans (35). CXCR6 is a chemokine receptor whose transmembrane ligand, CXCL16 is constitutively expressed on liver sinusoidal endothelial cells, where it provides anti-apoptotic signals to CXCR6^+^ liver resident NK cells and NK-T cells (62). We also demonstrated that, in mice and humans, NK cells are recruited to sites of antigen re-challenge and are phenotypically similar to tissue-resident NK cells (CD56^high^, CXCR6^+^, NKG2D^+^, CD69^+^, CD16^low^, T-bet^low,^ and Eomes^high^) (35, 60–63). Also, a small fraction of these CXCR6^+^ NK cells circulate in mice and humans (23, 36, 62). While CXCR6 is essential for memory NK cell survival, the mechanisms of antigen recognition and longevity for this memory NK cell subset remain to be elucidated (35, 60–63). While NKG2C is encoded by a germline-encoded and epigenetically regulated receptor, genomic modifications may underly non-CMV-specific NK memory mediated by CXCR6^+^ NK cells (64). These differences suggest NKG2C^+^ and CXCR6^+^ NK cells to be distinct in their adaptive immune mechanisms, perhaps as discrete NK cell subsets with unique and stable phenotypes and effector functions. However, this has not yet been investigated.

We hypothesize that NKG2C^+^ and CXCR6^+^ NK cells are distinct NK cell subsets with unique and stable phenotypes and effector functions, even during HCMV viremia. To test our hypothesis, we collected monthly PBMC samples from a cohort of 21 pediatric BMT/HSCT recipients that included both CMV-positive and CMV-negative subjects (1-15 years of age), starting one-month post-transplant. We lacked access to healthy pediatric control samples, so we used healthy adult (27-62 years of age) controls for comparison at each experimental time point. We performed in-depth analyses of the frequencies, phenotypic and functional markers of cellular differentiation (less differentiated CD56^bright^ vs. more differentiated CD56^dim^ NK cells), and NK cell subsets (NKG2C^+^CXCR6^–^ “NKG2C^+^”, NKG2C^–^CXCR6^+^ “CXCR6^+^, NKG2C^+^CXCR6^+^, and NKG2C^–^CXCR6^–^, over the course of a year. This approach allowed us to determine if NKG2C^+^ and CXCR6^+^ NK cells replenish as distinct NK cell subsets post-transplant in a setting where CMV is likely to reactivate, and to determine each subsets phenotypic characteristics and functional capabilities. Further, the reconstitution of NKG2C^+^ and CXCR6^+^ NK cell subsets could be interpreted in the context of the overall NK cell replenishment in pediatric transplant patients.

As hypothesized, we found that post-transplant CXCR6^+^, NKG2C^+^, and NKG2C^–^CXCR6^–^ NK cells are distinct NK cell subsets with unique phenotypic and functional attributes. Interestingly, NKG2C and CXCR6 are generally not co-expressed on NK cells following HSCT, regardless of the patient’s HCMV status, resulting in the absence of NKG2C^+^CXCR6^+^ NK cells in both HSCT pediatric recipients and healthy adult donors. Surprisingly, NKG2C–CXCR6– NK cells, rather than NKG2C^+^ NK cells, made up most NK cells post-transplant, even in transplant recipients with HCMV viremia. In contrast to NKG2C^+^ NK cells, CXCR6^+^ NK cells were less differentiated in phenotype, highly proliferative, and produced IFN-γ and TNF*α*. Additional functional studies revealed post-transplant NK to be cytotoxic and responsive to IL-2 stimulation. Our findings contribute to our understanding of post-transplant NK cell development and its implications for human health.

## Methods

### Patients, Samples, and Ethical Statement

Twenty-one patients with differing transplant indications (hematological malignancies, hemoglobinopathies, immune deficiencies, and bone marrow failure) were included in the study. Informed consent was obtained from the transplant recipient or their parent to participate in this study and from the healthy adult control donors, approved by the Baylor College of Medicine Institutional Review Board under IRB number H-35436, and study procedures were performed according to the principles of the declaration of Helsinki. Of the 21 transplant recipients, five received peripheral blood stem cells, one umbilical cord blood-derived stem cells, and fifteen bone marrow-derived stem cells. Peripheral blood samples derived from our cohort were collected monthly after transplantation. While 21 pediatric patients were enrolled, samples could not be collected from all participants at all time points due to the expected survival rate of pediatric transplant recipients and lack of retention due to the geographical relocation of some patients during the study year. As age-matched (pediatric) healthy control samples were not available, N=8-10 healthy adult donors (27-62 years of age) were evaluated instead. Patient diagnoses before transplant included B-cell acute lymphoblastic leukemia (N=5), severe aplastic anemia (N=3), acute myeloid leukemia (N=3), sickle cell disease (N=2), myelodysplastic syndrome associated with monosomy (N=7), chronic active EBV with hemophagocytic lymphohistocytosis (N=1), congenital dyserythropoeitic anemia (N=1), chronic granulomatous disease (N=1), beta thalassemia (N=1), T-cell ALL (N=1), and biphenotypic leukemia (N=1). N=5 patients received peripheral blood derived HSC, N=1 received umbilical cord blood-derived HSC, and N=15 patients received bone marrow derived HSC for their transplantation.

### KLRG2 Copy Number Variation of HSCT Patients

Copy number variation of the Killer Cell Lectin Like Receptor C2 (KLRC2) gene, encoding the NKG2C activating receptor, was assessed using Polymerase Chain Reaction. DNA from the following cell lines were included as controls: heterozygous HEK293T, homozygous null K562, and wild type 8866. KLRG2 deletion mutants generate a 411-base pair (bp) product, 201 bp products indicate wild-type KLRG2 gene products, and if both products are present, then samples are considered heterozygous for KLRG2. A single patient with an NKG2C-deletion (**Supplemental Figure 1**) was excluded from our flow cytometry analyses as this patient would not express NKG2C-protein and as such, subsets of NKG2C+ NK cells could not be identified.

HCMV DNA detection in sera from transplant patients was performed weekly by the Texas Children’s Hospital’s Clinical laboratory based on a published protocol (65).

### Isolation of Peripheral Blood Mononuclear Cells (PBMCs)

PBMC were separated using Ficoll-Paque™ PLUS (GE) gradients, frozen and subsequently thawed for flow cytometry. Only fresh (never frozen) PBMCs were used for functional assays shown in **Figure 6**.

### Determination of NKG2C Copy Numbers by Polymerase Chain Reaction (PCR)

NKG2C copy-number is a predictor for HCMV reactivation, and patients with a low copy-number have a higher probability of hCMV reactivation (45, 46, 50, 52). Copy-number variation of the *NKG2C* gene was assessed using a single-tube PCR reaction with two pairs of oligonucleotides per DNA template following a published protocol (66), using the following primers: NKG2C deletion Forward (ACTCGGATTTCTAT TTGATGC), NKG2C deletion Reverse (ACAAGTGATGT ATAAGAAAAAG), NKG2C wild type Forward (CAGTGTGGATCTTCAATG), NKG2C wild type Reverse (TTTAGTAATTGTGTG CATCCTA). DNA from the following cell lines were included as controls: heterozygous HEK293T (embryonic kidney), homozygous null K562 (Erythroleukaemia), and wild type RPMI 8866 (B lymphoblastoma) (66). *NKG2C* deletion mutants generate a 411-base pair (bp) product, 201-bp products indicate wild-type *NKG2C* products, and if both products are present, then samples are considered heterozygous for *NKG2C.*

### Flow Cytometry Staining

PBMCs were thawed, washed, and stained for extracellular markers. According to the manufacturer’s instructions, cells for intracellular and intra-nuclear markers were fixed and permeabilized using the FoxP3 permeabilization buffer kit (Tonbo Biosciences, San Diego, CA, USA). Multi-parametric analyses were performed on a 5-laser BD Fortessa (Becton, Dickinson, and Company, Franklin Lakes, NJ, USA), and data were analyzed using FlowJo software. Human natural killer cells were identified as single live cells that expressed CD45 and CD56 but not CD3. Antibodies, clone names, and vendor information can be found in **Supplemental Table 1**. Two panels examining developmental, maturation, tissue residency, and functional markers were used (**Supplementary Table 1**). **Panel 1:** CD45, CD3, CD56, CXCR6, NKG2C, CD57, NKG2A, NKG2D, T-BET, EOMES, TIM-3, CD16, CD94, CD62L; **Panel 2:** CD45, CD3, CD56, CXCR6, NKG2C, CD49E, CD27, PERFORIN, IFN-γ, TNF-α, Ki-67, KIR2DL2/DL3, CD69, GRANZYME B, TRAIL. The analysis of DNAM-1 expression on NK cells was not possible as we did not detect any DNAM-1 expression in any samples, including on NK cells collected from healthy adult control PMBCs, indicating a problem with the antibody staining. Also noteworthy is that we did not stimulate PBMCs *in vitro* before their analysis to preserve their *ex vivo* profile, including any proliferation induced by post-transplant homeostatic expansion and effector functions based on viral reactivation rather than artificial stimuli. Sufficient cell numbers were available for the statistical evaluation of NK cell-expressed markers even when rare populations were analyzed. On average, 2,747 NKG2C^+^ and 548 CXCR6^+^ NK cells were analyzed per sample, with samples comprised of mostly NKG2C^–^CXCR6^–^ NK cells. For flow cytometry, samples were analyzed together to avoid batch effects.

### Data Analysis and Software

FCS files were transferred to FlowJo software (TreeStar). The NK cells were gated on physical properties (live and single cells) and combined with lineage-specific markers (CD45^+^ CD3^–^ CD56^+^). Fluorescence minus one (FMO) staining was used in every experiment to determine positive and negative populations, and compensation was performed individually for each experiment using OneComp eBeads (eBioscience).

### Functional Assay (Chromium Release Assay)

NK cell cytotoxicity activity of fresh PBMCs from BMT/HSCT patients against the K562 erythroleukemia target cell line was assessed using a standard (51)Chromium (Cr) release assay. K562 cells were cultured in RPMI 1640 medium supplemented with 10% FBS, and L-glutamine (R10) and incubated at 37°C with 5% CO_2_. K562 target cells were labeled with 100μCi (51)Cr (Na_2_CrO_4_) (Perkin-Elmer) per 10^6^ cells (51).Cr -labeled K562 target cells were incubated with PBMCs at the indicated effector to target ratios in 96-well round-bottom plates and incubated for 4 hours at 37°C, 5% CO_2_. Targets and effectors were either incubated in R10 media alone or in the presence of 1,000 IU/mL IL-2. Following incubation, one row of target cells was lysed with 1.0% IGEPAL (Sigma) for total release, and plates were centrifuged at 300Xg for 10 minutes with no brake. Supernatants were harvested and transferred to a LUMA plate (Perkin Elmer) and dried overnight. Plates were counted on a TopCount NLT gamma detector (Perkin Elmer). Spontaneous release of (51)Cr into the supernatant by target cells alone was used as background and subtracted from all experimental values. Percent specific lysis = (Experimental cpm – spontaneous release cpm)/Total cpm – spontaneous cpm) X 100.

### Statistical Analysis

**Figure 1:**
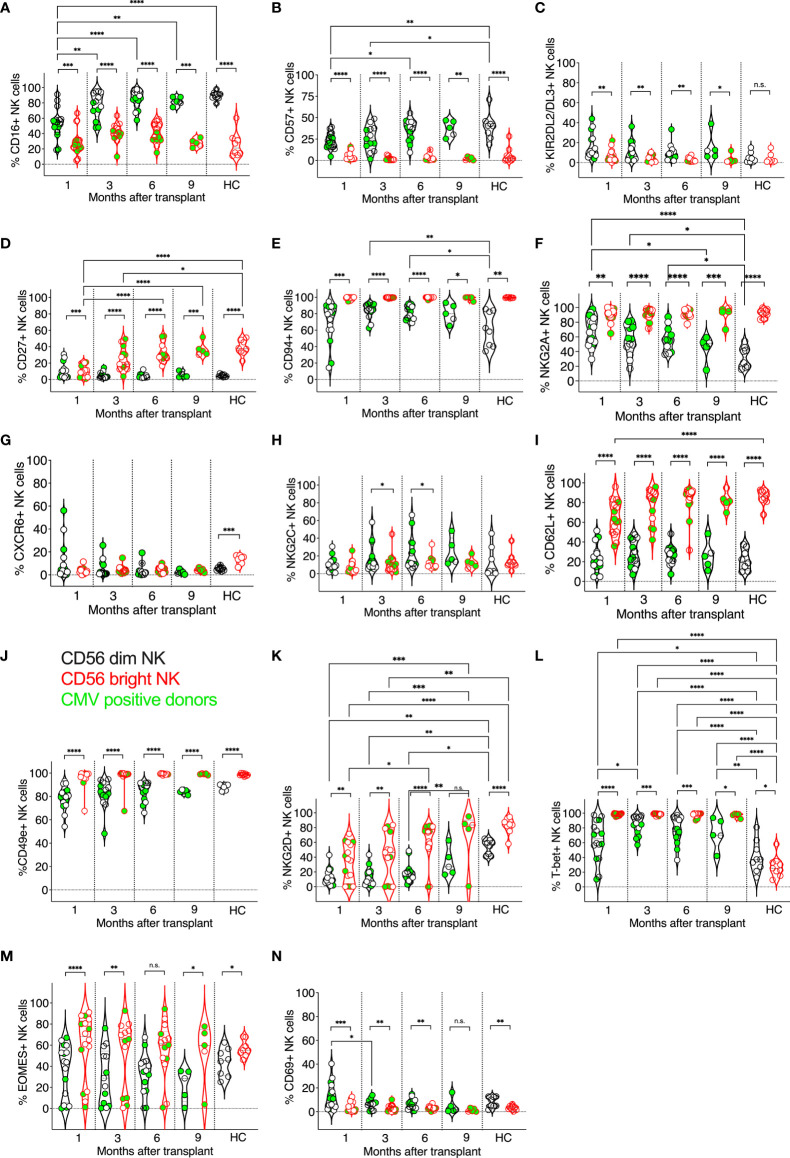
Expression kinetics of developmental markers on CD56^dim^ and CD56^bright^ NK cells post-transplant. The frequencies of developmental markers **(A–N)** were determined for each patient’s circulating NK cells at indicated time points using multiparameter flow cytometry. Circulating healthy adult donor NK cells were also analyzed as a control. Each data point represents a single transplant recipient or healthy adult control donor (HC). Data points in black represent CD56^dim^ NK cells; data points in red represent CD56^bright^ NK cells. Data points with a neon-green fill color represent patients positive for CMV up to and at the analysis time as determined by quantitate PCR. *p < 0.05; **p < 0.005; ***p < 0.0005; ****p < 0.0005; Not significant (n.s.).

**Figure 2:**
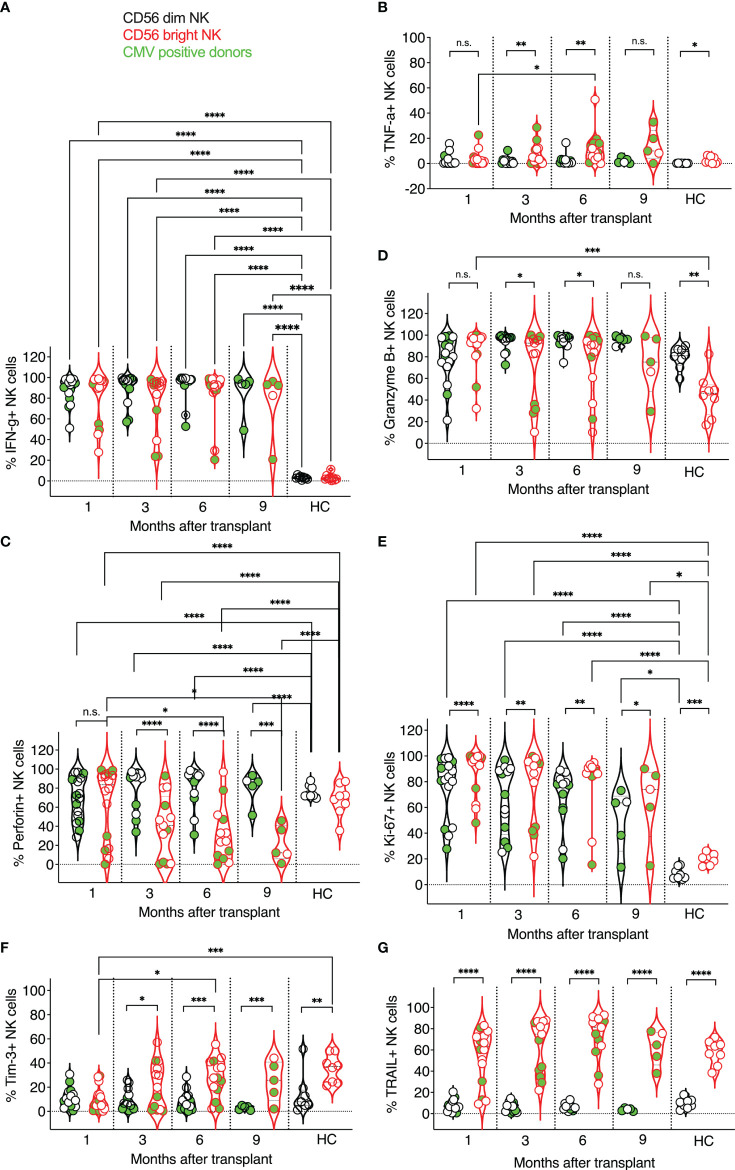
Expression kinetics of functional markers on CD56^dim^ and CD56^bright^ NK cells post-transplant. The frequencies of functional markers **(A–G)** were determined for each patient’s circulating NK cells at indicated time points using multiparameter flow cytometry. Circulating healthy adult donor NK cells were also analyzed as a control. Each data point represents a single transplant recipient or healthy adult control donor (HC). Data points in black represent CD56^dim^ NK cells; data points in red represent CD56^bright^ NK cells. Data points with a neon-green fill color represent patients positive for CMV up to and at the analysis time as determined by quantitate PCR. *p <0.05; **p < 0.005; ***p < 0.0005; ****p <0.0005; Not significant (n.s.).

**Figure 3:**
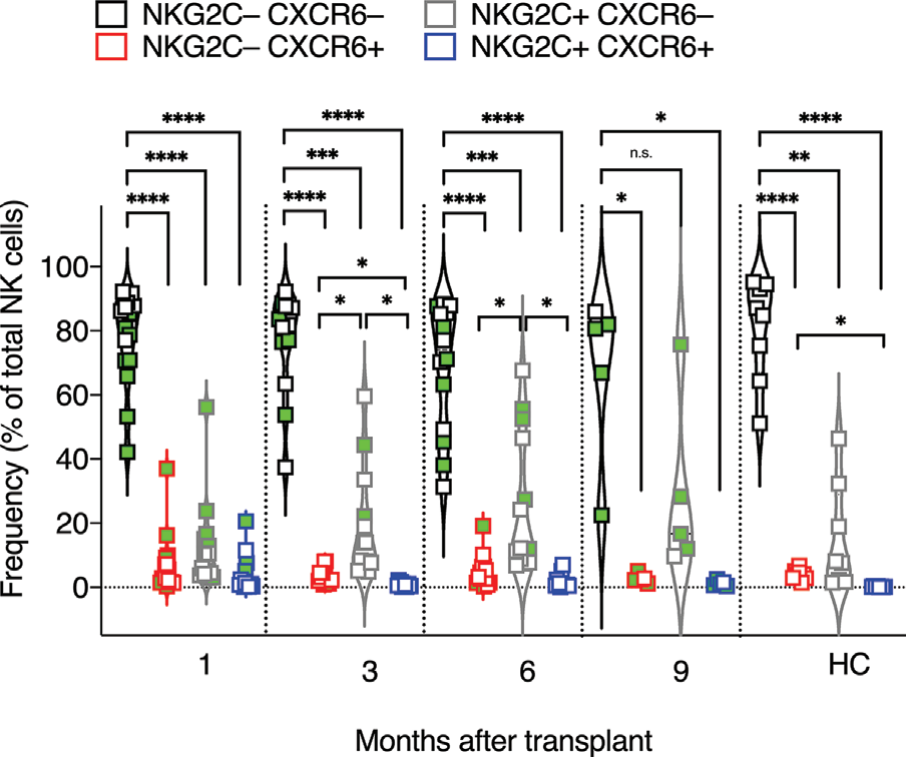
Frequencies of CXCR6^+^, NKG2C^+^, CXCR6^–^NKG2C^–^, and CXCR6^+^NKG2C^+^ NK cells. The frequencies of CXCR6^+^, NKG2C^+^, CXCR6^–^NKG2C^–^, and CXCR6^+^NKG2C^+^ NK cells were determined for each patient’s circulating NK cells at indicated time points using flow cytometry. Circulating healthy adult donor NK cells were also analyzed as a control. Each data point represents a single patient or healthy adult control donor (HC). Data points with a neon-green fill color represent patients positive for CMV up to and at the analysis time as determined by quantitate PCR. *p <0.05; **p < 0.005; ***p < 0.0005; ****p <0.0005; Not significant (n.s.).

**Figure 4:**
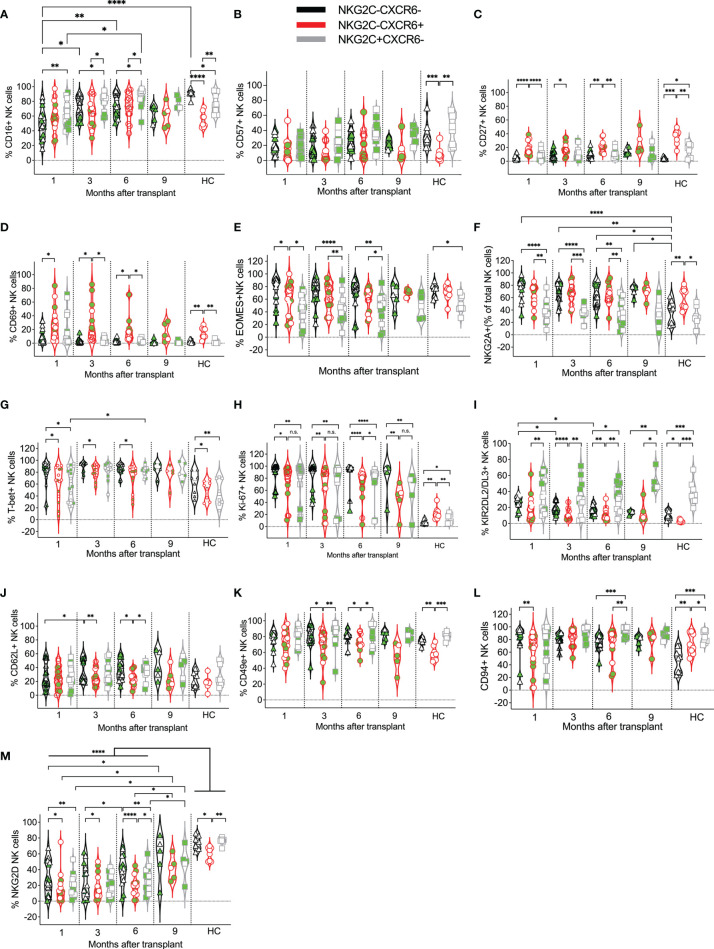
Expression kinetics of developmental markers on CXCR6^–^NKG2C^–^, CXCR6^+^, and NKG2C^+^ NK cells post-transplant. The frequencies of the indicated developmental markers **(A–M)** were determined for each patient’s CXCR6^–^NKG2C^–^, CXCR6^+^, and NKG2C^+^ NK cells at the indicated time points. Data points in black represent CXCR6^–^NKG2C^–^ NK cells; data points in red represent CXCR6^+^ NK cells, and data points in gray represent NKG2C^+^ NK cells. Circulating healthy adult donor NK cells were also analyzed as a control. Each data point represents a single transplant recipient or healthy adult control donor (HC). Data points with a neon-green fill color represent patients positive for CMV up to and at the analysis time as determined by quantitate PCR. *p < 0.05; **p < 0.005; ***p < 0.0005; ****p < 0.0005; Not significant (n.s.).

**Figure 5:**
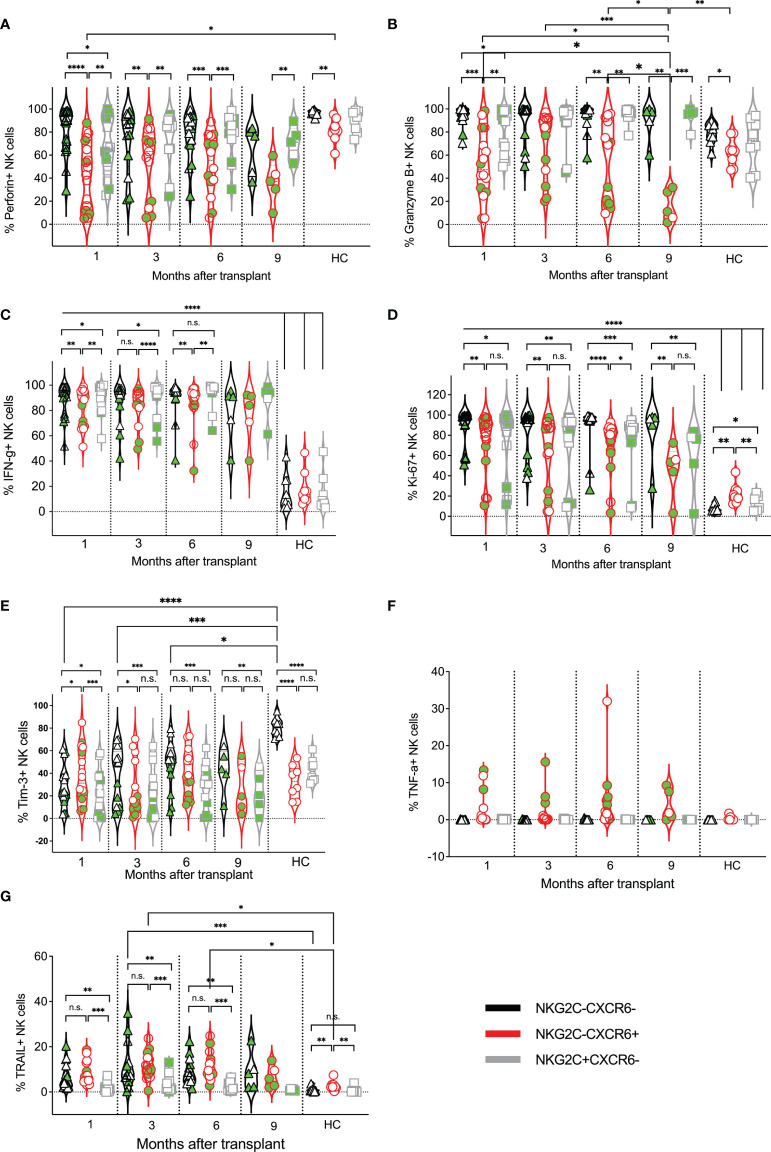
Expression kinetics of functional markers on CXCR6^–^NKG2C^–^, CXCR6^+^, and NKG2C^+^ NK cells post-transplant. The frequencies of indicated functional markers **(A–G)** were determined for each patient’s CXCR6^–^NKG2C^–^, CXCR6^+^, and NKG2C^+^ NK cells at indicated time points. Data points in black represent CXCR6^–^NKG2C^–^ NK cells; data points in red represent CXCR6^+^ NK cells, and data points in gray represent NKG2C^+^ NK cells. Circulating healthy adult donor NK cells were also analyzed as a control. Each data point represents a single transplant recipient or healthy adult control donor (HC). Data points with a neon-green fill color represent patients positive for CMV up to and at the analysis time as determined by quantitate PCR. *p < 0.05; **p < 0.005; ***p < 0.0005; ****p < 0.0005; Not significant (n.s.).

**Figure 6:**
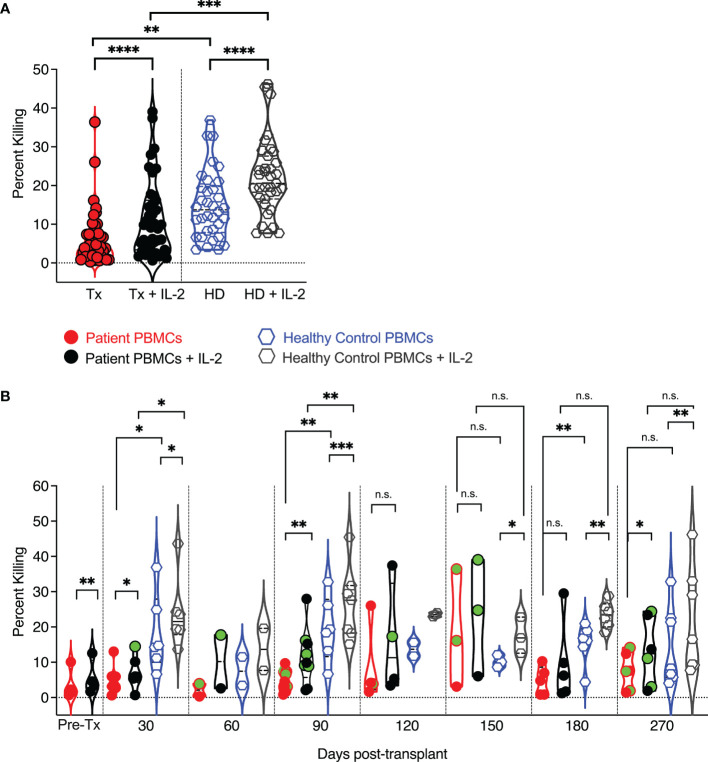
Cytolytic potential of pediatric transplant patients and IL-2 responsiveness vs. human adult donor NK cells. PBMCs were added to (51) Cr labeled K562 target cell at an effector to target ratio of 25:1. After four hours of co-incubation, supernatants were harvested and counted in a gamma counter to assess (51)Cr release as a measure of target lysis. At each time point, circulating healthy adult control donor NK cells were also analyzed. Each data point represents a single transplant recipient or healthy adult control donor (HC). Data points with a neon-green fill color represent patients positive for CMV up to and at the analysis time as determined by quantitate PCR. **(A)** Pooled analysis of all time points and comparison of adult patient and healthy adult donor NK cell cytotoxic capabilities. **(B)** Cytolytic potential of pediatric transplant patients vs. human adult donor NK cells and IL-2responsiveness at each examined time point post-transplant and in comparison to healthy adult control NK cells assessed at each time point. *p < 0.05; **p < 0.005; ***p < 0.0005; ****p < 0.0005; Not significant (n.s.).

A paired t-test was used for between two-group comparisons (**Figures 1**, **2**, at each time point and for healthy donors to compare the expression of the indicated marker on CD56^dim^ vs. CD56^bright^ NK cells). One-way ANOVA with Tukey’s multiple comparisons test was used to compare the study endpoints among three or more groups (**Figures 1**, **2**, to compare CD56^dim^ and CD56^bright^ NK cells longitudinally and with healthy donor control NK cells, and **Figures 3**–**5**: to compare three (**Figures 4**, **5**) or more (**Figure 3**) NK cell subsets at each time point post-transplant and to compare each subset longitudinally and with healthy donor control NK cells). A Welch’s t-test was used to compare the killing capabilities of circulating healthy donor NK cells vs. post-transplant patient NK cells (**Figure 6**). For the statistical determination of IL-2 sensitivity, a paired t-test was used (**Figure 6**). The statistical significance (p-values) are as follows: *p<0.05; **p<0.005; ***p<0.0005; ****p<0.0005. Graphs and statistical analyses were performed with GraphPad Prism 9 (GraphPad Software, La Jolla, CA, USA).

## Results

### Developmental Phenotypes of CD56^dim^ vs. CD56^bright^ NK Cells

We examined the frequencies, phenotypic and functional markers of CD56^dim^ vs. CD56^bright^ NK cells, and NKG2C^+^ vs. CXCR6^+^, NKG2C^+^CXCR6^+^, and NKG2C^–^CXCR6^–^ NK cell subsets in PBMCs from a cohort of 21 pediatric transplant recipients. In our analyses, we included both CMV-positive (N=9) and CMV-negative subjects (N=11) (both 1-15 years of age). We analyzed NK cells phenotypically at one month, three, six, and nine months post-transplant and compared results to those of healthy adult controls (N=8-10; 27-62 years of age). As expected, we found NK cells to be abundant in human PBMCs one-month post-transplant. They declined in frequency gradually, presumably due to the expansion of other immune cell types, such as T cells (**Supplemental Figure 2**).

We compared the expression of developmental markers on donor-matched CD56^bright^ and CD56^dim^ NK cell subsets, which in PBMCs are classically considered less vs. more differentiated NK cells (67–70) (**Figure 1**). As expected, when comparing CD56^bright^ and CD56^dim^ NK cell subsets from post-transplant patients and healthy controls, significant differences in marker expression was observed: more CD56^dim^ NK cells expressed the activating receptor FcγRIII (CD16) compared to CD56^bright^ NK cells, albeit CD56^dim^ post-transplant NK cells initially expressed CD16 at lower levels than CD56^dim^ healthy control NK cells and gradually increased their CD16 expression post-transplant (**Figure 1A**). Also, more CD56^dim^ post-transplant NK cells expressed the terminal differentiation marker CD57 and inhibitory KIR2DL2/DL3 than donor-matched CD56^bright^ NK cells (**Figures 1B, C**). In contrast, CD56^bright^ NK cells expressed the developmental marker CD27 more frequently than CD56^dim^ NK cells, and its expression became indistinguishable from healthy adult controls by six months post-transplant (**Figure 1D**). Nearly all CD56^bright^ NK cells expressed CD94, which forms heterodimers with NKG2A and NKG2C. also, CD94 expression was higher on CD56^bright^ NK cells at three and six-month post-transplant compared to healthy adult controls but normalized at 9 months post-transplant (**Figure 1E**). CD94 expression was more variable on CD56^dim^ post-transplant NK cells but equal to that of healthy controls at all time points (**Figure 1E**). The inhibitory receptor NKG2A was detected on both CD56^bright^ and CD56^dim^ post-transplant NK cells. As expected, significantly more CD56^bright^ NK cells expressed NKG2A than post-transplant CD56^dim^ NK cells, however, those expressed higher levels of NKG2A compared to CD56^dim^ NK cells of healthy controls, especially early post-transplant (**Figure 1F**). When comparing CD56^bright^ vs. CD56^dim^ NK cells in transplant patients and healthy adult controls, we found no significant difference in the expression of CXCR6 on post-transplant CD56^bright^ vs. CD56^dim^ NK cells, however, in healthy controls, significantly more CD56^bright^ NK cells expressed CXCR6 compared to CD56^dim^ NK cells (**Figure 1G**). In contrast, NKG2C was not differentially expressed on CD56^bright^ vs. CD56^dim^ NK cells in healthy controls but was elevated on CD56^dim^ NK cells compared to donor-matched CD56^bright^ NK cells at three and six months post-transplant (**Figure 1H**). In patients and healthy adult control donors, CD56^bright^ NK cells were also significantly more likely to express CD62L (L-selectin) (**Figure 1I**), CD49e (integrin alpha 5) (**Figure 1J**), and, surprisingly, the activating receptor NKG2D (**Figure 1K**), and the transcription factor T-bet (**Figure 1L**), compared to CD56^dim^ NK cells. While T-bet was expressed by more post-transplant NK cells of either subset, NKG2D was significantly less frequently expressed compared to healthy donor control NK cells, especially in CD56^dim^ post-transplant NK cells (**Figure 1K**). As expected, more CD56^bright^ post-transplant NK cells expressed the transcription factor EOMES (**Figure 1M**), compared to donor-matched CD56^dim^ NK cells, both after transplant and in healthy adult controls. The numbers of circulating CD69^+^ NK cells were generally low (**Figure 1N**); however, both in post-transplant and healthy donor controls, more CD56^dim^ NK cells expressed CD69 than CD56^bright^ NK cells.

Prior work by us and others revealed that tissue-resident CXCR6^+^ NK cells generally display a less differentiated phenotype (CD56^bright^, CD69^+^, CD49e^−^, CD16^dim^, CD57^dim^, KIR^dim^, and NKG2A^+^) and are enriched in the CD56^bright^ population (21, 23, 36). However, in contrast to tissue-resident NK cells, we did not observe statistically significant differences in the frequency of CXCR6^+^ NK cells when comparing CD56^dim^ vs. CD56^bright^ post-transplant NK cells (**Figure 1I**). Both CXCR6 and NKG2C were expressed by CD56^dim^ vs. CD56^bright^ healthy control NK cells. Further, the expression of CXCR6, NKG2C, KIR2DL2/DL3, EOMES, CD69, and CD62L and CD49e on CD56^bright^ and CD56^dim^ NK cell subsets was not altered upon transplantation compared to healthy controls. Of note, we also did not observe significant differences in the frequency of the afore-analyzed NK cell-expressed markers when comparing CMV positive (neon green filled symbols) vs. negative transplant recipients or NKG2C heterozygous vs. NKG2C wild type NK cells (not shown).

### Markers of NK Cell Effector Functions Expressed by CD56^dim^ and CD56^bright^ NK Cells

Next, we examined the effector functions of CD56^dim^ vs. CD56^bright^ NK cells at one month, three, six, and nine months post-transplant, and compared these data to circulating healthy adult control NK cell subsets (**Figure 2**). We evaluated NK cells *ex vivo* to preserve the impact of post-transplant NK cell expansion and *in vivo* NK cell activation by infections that commonly occur post-transplant. IFN-γ production was indistinguishable between CD56^dim^ and CD56^bright^ NK cells post-transplant (**Figure 2A**) and significantly higher in transplant recipients than in healthy adult controls, in which NK cells do not undergo lymphopenic expansion and infections are presumably more controlled (**Figure 2A**). Interestingly, we observed little TNF-α production in either subset (**Figure 2B**), albeit some patient’s CD56^bright^ NK cells produced TNF-α at each time point post-transplant. As expected, perforin expression was more frequent in CD56^dim^ NK cells than in CD56^bright^ NK cells. Also, perforin expression varied in both CD56^dim^ and CD56^bright^ NK cells and was less prevalent in post-transplant NK cells than in healthy adult controls (**Figure 2C**). Granzyme B expression was also higher in CD56^dim^ post-transplant NK cells and like that of healthy adult donors for both NK cell subsets (**Figure 2D**). Both CD56^dim^ and CD56^bright^ NK cell subsets proliferated vigorously compared to healthy adult NK cells, as indicated by their high expression of Ki-67, with a higher frequency of proliferating CD56^bright^ NK cells. This intense proliferation is likely due to the robust homeostatic expansion in transplant recipients (**Figure 2E**). Interestingly, Tim 3-expression was more frequently observed on CD56^bright^ NK cells, resembling healthy adult control donor-derived NK cells at three-month post-transplant (**Figure 2F**). Interestingly, the expression of TNF-related apoptosis-inducing ligand (TRAIL) on post-transplant NK cells was also like that of healthy adult control NK cells and stable throughout the length of this study (**Figure 2G**). TRAIL was expressed early on reconstituting NK cells, with more than half of the CD56^bright^ NK cell subpopulation expressing TRAIL one month post-transplant. In contrast, few CD56^dim^ NK cells expressed TRAIL in post-transplant or healthy control donors (**Figure 2G**). Our findings are interesting in the context of data presented at an American Society of Hematology meeting. The frequency of circulating cytotoxic CD56^bright^ TRAIL-expressing NK cells was linked to a better outcome after allogeneic stem cell transplantation (71).

### Phenotypes of CXCR6^+^, NKG2C^+^ NK Cells, and CXCR6^–^NKG2C^–^ NK Cells

We next examined the expression of NKG2C vs. CXCR6 on post-transplant NK cells, as both markers have been implicated in NK memory responses to CMV (47, 49, 51, 61) and non-CMV viral antigens (35, 72), and because we speculated that NKG2C^+^ and CXCR6^+^ NK cells are distinct NK cell subsets with unique and stable phenotypes and effector functions, even during HCMV viremia. Interestingly, most post-transplant NK cells expressed neither NKG2C nor CXCR6, including in transplant recipients where HCMV reactivated post-transplant. The frequency of CXCR6^–^NKG2C^–^ NK cells was statistically significantly higher at all time points post-transplant and in healthy control donors (**Figure 3**). CXCR6^+^ NK cells and NKG2C^+^NK cells were also detected. Interestingly, CXCR6^+^NKG2C^+^ NK cells were scarce after the first four weeks post-transplant and were too few to be assessed for phenotype or function, even upon HCMV reactivation (**Figure 3**). We conclude that CXCR6^+^ NK cells and NKG2C^+^NK cells are distinct NK cell subsets.

We next investigated the phenotypes of CXCR6^–^NKG2C^–^, CXCR6^+^, and NKG2C^+^ NK cells at each post-transplant time point and compared them to healthy adult control NK cells. In healthy donors, CD16 was most commonly expressed by CXCR6^–^NKG2C^–^, followed by NKG2C^+^ NK cells, and only about one half of CXCR6^+^ NK cells expressed CD16 (**Figure 4A**). In contrast, in post-transplant patients, CD16 was equally expressed by CXCR6^–^NKG2C^–^ and CXCR6^+^ NK cells, and more NKG2C^+^ NK cells expressed CD16 than CXCR6^+^ or CXCR6^–^NKG2C^–^ NK cells. CD57-expression was similar across all NK cell subsets and post-transplant time points (**Figure 4B**). CD57 was also expressed by healthy control CXCR6^–^NKG2C^–^ and NKG2C^+^ NK cells, while few CXCR6^+^ NK cells expressed this terminal differentiation marker (**Figure 4B**). In contrast, CD27 was expressed on most healthy adults’ CXCR6^+^ NK cells compared to NKG2C^+^ and CXCR6^–^NKG2C^–^ NK cells. This trend was also noticeable in post-transplant patients (**Figure 4C**). Also, CD69 was expressed by significantly more CXCR6^+^ NK cells than NKG2C^+^ and/or CXCR6^–^NKG2C^–^ NK cells in healthy control donors and on post-transplant NK cells at most time points (**Figure 4D** **)**, perhaps indicating that CXCR6^+^ NK cells are generally tissue-resident NK. More CXCR6^–^NKG2C^–^ and CXCR6+ NK cells expressed the T-box transcription factor Eomes (**Figure 4E**) and the inhibitory receptor NKG2A (**Figure 4F**) post-transplant than NKG2C^+^ NK cells. Similar differences were observed in healthy adults. T-bet expression was most frequent in CXCR6^–^NKG2C^–^ NK cells and higher in CXCR6+ NK cells in pediatric post-transplant patients and healthy adult controls (**Figure 4G**). Interestingly, CXCR6^–^NKG2C^–^ NK cells proliferated the most, albeit CXCR6^+^ and NKG2C^+^ NK cells also showed high levels of proliferation post-transplant. In contrast, as expected, proliferation was low in healthy control donor NK cells and highest in CXCR6^+^ NK cells (**Figure 4H**).

As previously reported, NKG2C^+^ NK cells more frequently expressed KIR2DL2/DL3 than CXCR6^+^ NK cells and CXCR6^–^NKG2C^–^ NK cells at later time points post-transplant, as well as in healthy adults (**Figure 4I**), resembling NKG2C^+^ memory NK cells in phenotype. Few differences were observed in CD62L (**Figure 4J**) expression both in post-transplant and healthy control NK cells, with a slightly lower expression of these markers on CXCR6^+^ NK cells, albeit these differences were not always statistically significant. The frequency of CD49e and CD94-expressing NK cells was high across all NK cell subsets; however, more NKG2C^+^ NK cells expressed these markers than CXCR6^+^ and CXCR6^–^NKG2C^–^ NK cells (**Figures 4K, L**), albeit these differences were not always statistically significant. Interestingly, compared to healthy adult NK cells, NKG2D-expressing NK cells were reduced in all three post-transplant NK cell subsets compared to healthy adult donor NK cells. NKG2D-expression was initially reduced progressively acquired post-transplant, as previously reported (73), albeit it never quite reached the level of expression seen in healthy adult NK cells (**Figure 4M**).

### Effector Functions of CXCR6^–^NKG2C^–^, CXCR6^+^, and NKG2C^+^ NK Cells

We next compared the effector functions of CXCR6^–^NKG2C^–^, CXCR6^+^, and NKG2C^+^ NK cells to each other and adult human circulating NK cells. Interestingly, the frequency of perforin-expressing NK cells was quite varied and relatively low in several post-transplant patients (**Figure 5A**). No differences in perforin or granzyme B expression were observed in healthy adult NK cells, regardless of the subset (**Figure 5B**). In contrast, fewer post-transplant CXCR6^+^ NK cells expressed perforin than CXCR6^–^NKG2C^–^ and NKG2C^+^ NK cells. Similarly, the frequency of granzyme B-expressing NK cells was lowest in post-transplant CXCR6^+^ NK cells, although expression varied. Also, we observed a significant drop in the frequency of granzyme B-expressing CXCR6^+^ NK cells in transplant recipients at 9-months post-transplant. This is interesting as the expression of cytolytic enzymes generally increases with lymphocyte maturation, and perforin is typically co-expressed with granzymes A and B (74). However, CXCR6^+^ NK cells do not follow these previously reported phenotypes.

Additional effector function molecules were evaluated. IFN-γ producing and proliferating (Ki-67-expressing) NK cells were significantly increased in all three NK cell subsets post-transplant compared to healthy controls, presumably due to the expansion of developing NK cells in transplant recipients and their activation by infections (**Figures 5C, D**). However, a few transplant recipients did not have proliferating NK cells, especially in the CXCR6^+^ and NKG2C^+^ subsets. The frequency of Tim-3 expressing NK cells was also quite varied in all three post-transplant subsets and, for the CXCR6^–^NKG2C^–^ NK cell population, generally lower than that of healthy adults (**Figure 5E**). Surprisingly, in healthy adult donors, more than half of CXCR6^–^NKG2C^–^ NK cells expressed Tim-3, contrary to donor matched CXCR6^+^ and NKG2C^+^ NK cell subsets, by which Tim-3 expression was observed in less than half of NK cells. Finally, TNF-*α* expression was generally low across all NK cell subsets in transplant recipients and healthy adult controls and restricted to CXCR6^+^ patient-derived NK cells. (**Figure 5F**). In contrast, the frequency of TRAIL-expressing NK cells was higher on all post-transplant NK cell subsets than circulating healthy human control NK cells and significantly higher in CXCR6^+^ NK cells than NKG2C+NK cells (**Figure 5G**). A summary of the frequency of expression for all markers analyzed in **Figures 4**, **5** is shown as a heat map in **Supplemental Figure 3** for easier comparison.

### NK Cell Killing Is Similar Between Pediatric Patients and Healthy Adult Controls

Given the differences in granzyme and perforin expression between pediatric post-transplant and healthy adult control circulating NK cells, we next tested whether their cytotoxic capability was distinct. We performed chromium release assays and assessed the killing of K562 target cells by pediatric post-transplant NK cells at one month, two-, three-, four-, five, six-, and nine months post-transplant (**Figure 6**). At each time point, the cytotoxic capability of healthy adult control NK cells was also examined. Both pediatric post-transplant and healthy adult control NK cells killed K562 target cells and were sensitive to IL-2 stimulation, albeit healthy donor control NK cells killed more K562 target cells than post-transplant NK cells (**Figure 6A**). Between five -and nine-months post-transplant, the cytolytic potential of patient-derived NK cells was like that of healthy donor-derived NK cells, and both sets of NK cells responded to IL-2 stimulation (**Figure 6B**). We were unable to isolate CXCR6^+^, NKG2C^+,^ and CXCR6^–^NKG2C^–^ NK cells to analyze them for potential differences in their cytotoxic capabilities, as pediatric PBMC samples are limited in volume.

## Discussion

HSCT is a life-saving therapy for patients with various diseases, including hematological malignancies, hemoglobinopathies, bone marrow failure, and immunodeficiencies (75). NK cell reconstitution and the developmental and functional capabilities of post-transplant NK cell subsets are poorly understood, especially in pediatric patients. Recently, memory NK cells that respond to vaccination and expand following infection with HCMV and other pathogens have been described in mice and humans (15, 17, 35, 44, 47, 49, 50, 56, 60–63, 72, 76). These NK cell subsets have important implications in host protection from infection, including in the transplant setting (45, 47, 48). However, whether CXCR6^+^ and NKG2C^+^ NK cells are discrete NK cell subsets with unique and stable phenotypes and effector functions has not yet been evaluated. Therefore, we performed in-depth immune phenotyping by multi-parametric flow cytometry and functional assays to investigate pediatric post-transplant NK cell developmental phenotypes and effector functions and compared results to healthy adult control NK cells. We focused on less and more differentiated NK cells and two memory NK cell populations, namely CMV-specific NKG2C^+^ vs. non-CMV-specific CXCR6^+^ NK cells.

Generally, CD56^bright^ and CD56^dim^ NK cells were phenotypically distinct, and CD565^bright^ NK cells were less differentiated than CD56^dim^ NK cells. Also, a subset of CD56^dim^ and CD565^bright^ NK cells expressed CXCR6 or NKG2C, contradicting the assumption that CXCR6+ NK cells are phenotypically like CD56^bright^ NK cells in peripheral blood. Noticeable differences between patient and healthy adult control NK cells were a lower expression of the activating receptor NKG2D post-transplant and a more activated phenotype, including high IFN-γ expression and significant proliferation of both CD56^bright^ and CD56^dim^ NK cells in patients. This phenotype is expected as NK cells expand in lymphopenic transplant recipients who experience a range of viral infections, including Epstein Barr virus, Human Herpesvirus-6, Adenovirus, BK virus, and Respiratory Syncytial Virus infections, or a combination of these. In contrast, the expression of CXCR6, NKG2C, KIR2DL2/DL3, EOMES, CD69, and CD62L and CD49e on CD56^bright^ and CD56^dim^ NK cell subsets was not altered upon transplantation compared to healthy controls.

Remarkably, while CXCR6^–^NKG2C^–^, CXCR6^+^, and NKG2C^+^ NK cells developed as early as one-month post-transplant, we did not find CXCR6^+^NKG2C^+^ NK cells in transplant patients, even in patients with HCMV disease. CXCR6^–^NKG2C^–^ NK cells shared characteristics with both NKG2C^+^ and CXCR6^+^ NK cells, albeit they were functionally more like NKG2C^+^ NK cells. NK cells already express CXCR6 during fetal development (23), and CXCR6-expression seems stable on NK cells (36), albeit it can be induced *in vitro* by cytokine stimulation (34). The regulation of *KLRC2* gene expression (encoding for NKG2C) is currently incompletely understood, and future work is needed to better understand the mechanisms underlying NK cell subset-specific CXCR6 and NKG2C expression and to elucidate whether CXCR6^–^NKG2C^–^ NK cells are distinct from or a precursor of the NKG2C^+^ NK cell subset. Surprisingly, in healthy adult donors, almost all CXCR6^–^NKG2C^–^ NK cells expressed Tim-3, contrary to donor-matched CXCR6^+^ and NKG2C^+^ NK cell subsets. This is interesting, as prior published work has implicated Tim-3 in the downregulation of NK cell-expressed T-bet and functional regulation of NKG2C^+^CD57^+^ double-positive NK cells (77). Specifically, *in vitro* Tim-3 blockade resulted in the release of cytokines, chemokines, and cytotoxic molecules in response to cytokine or activating receptor ligation (CD16) but did so only in stimulated NKG2C^+^CD57^+^ NK cells (77). In contrast, NKG2C-negative NK cells were unaffected by Tim-3 blockade (77). We found most CXCR6^–^NKG2C^–^ NK cells to express Tim-3, albeit we did not evaluate its expression functionally. Future work is needed to elucidate Tim-3’s role in NK cell functional subsets.

CXCR6^+^ NK cells were less likely to express perforin and especially granzyme B than CXCR6–NKG2C– and NKG2C+ NK cells and presented a significant reduction in granzyme B-expression 9-months post-transplant. Whether perforin and granzyme B were lower in transplant patients than in healthy controls because of continuous degranulation or post-transplant reconstitution will require further study. Even though CXCR6^+^ NK cells were phenotypically less differentiated than NKG2C^+^ NK cells, they were highly proliferative and produced significant amounts of IFN-γ. These characteristics resemble tissue-resident CXCR6^+^ NK cells and a rare subset of CXCR6^+^ NK cells found in healthy adult peripheral blood (15, 17, 21, 24, 36).

Few existing studies have examined NK cell reconstitution in pediatric HCT patients. A recent study by Stabile, Gismondi, and colleagues described a circulating and bone marrow resident CD56^low^CD16^low^CD25 ^low^CD127 ^low^CD122^high^ NK cell subset capable of significant INFγ production upon cytokine stimulation. These cells became cytotoxic over time and may play an essential role in the graft vs. leukemia effect (41). Another study by Roberto, Mavilio, and colleagues also evaluated an unconventional subset of anergic NKp46^low^D56^dim^CD16^neg^ NK cells in adult haploidentical HSCT recipients, whose cytotoxic defects could be reversed by NKG2A blockade, at least *in vitro*. We did not evaluate these populations in our study but instead focused on analyzing the distinct phenotypes and effector functions of CXCR6^+^ and NKG2C^+^ NK cell subsets. However, together, these findings justify the need to identify and evaluate NK cell functional subsets in health and disease so that novel therapeutic targets can be identified and evaluated.

NKG2C is encoded by the KLRC2 gene (66). We assessed the effects of *KLRC2* copy number variance on human post-transplant NK cell phenotypes and effector functions. We found that heterozygosity did not significantly modulate NK cell phenotypes or effector functions (not shown). NK cells from NKG2C-heterozygous transplant recipients were like those of wild-type transplant recipients. These data are interesting considering published reports hypothesizing that CMV reactivation increases morbidity and mortality in post-transplant patients and that NKG2C heterozygosity or deficiency may increase the incidence of CMV reactivation, CMV-dependent morbidity, and/or mortality (46). However, as our cohort is small, additional data is needed to investigate this discrepancy further. Of note, one transplant recipient who had a deletion of *NKG2C* was not included in the analysis. We were unable to identify NKG2C^+^ NK cells in this transplant recipient for our study. This transplant recipient did reactivate CMV and subsequently EBV but otherwise was indistinguishable in NK cell-expressed markers when analyzed in a non-NKG2C-specific manner (not shown). Notably, after transplantation, HSCT recipients experienced a range of viral infections, including Epstein Barr virus, Human Herpesvirus-6, Adenovirus, BK virus, and Respiratory syncytial virus infections, or a combination of these. However, our cohort is too small to conclude how specific viral infections other than CMV may impact NK cell phenotypes and effector functions.

Although the number of patients in our study is limited, which likely affected our ability to evaluate the 9-month post-transplant time point statistically, our data demonstrate the dynamic kinetics in acquiring developmental and functional capabilities of NK cell subsets post-transplant. Our data strongly suggest that NKG2C^+^ and CXCR6^+^ NK cells are distinct, and CXCR6^+^NKG2C^+^ NK cells are rare, even in CMV^+^ subjects. In contrast, single positive NK cells for both NKG2C and CXCR6 are present early post-transplant, with an expected and observed increase in NKG2C^+^ NK cells upon CMV-reactivation. Our data inform on the development of specific NK cell subsets and their functions post-transplant. Our findings may allow for the selection of superior NK cell functional subsets for improved immunotherapies to treat post-transplant viral infections and malignancy.

## Data Availability Statement

The original contributions presented in the study are included in the article/**Supplementary Material**. Further inquiries can be directed to the corresponding author.

## Ethics Statement

Informed consent was obtained from the transplant recipient or their parent to participate in this study and from the healthy adult control donors, approved by the Baylor College of Medicine Institutional Review Board under IRB number H-35436, and study procedures were performed according to the principles of the declaration of Helsinki. Written informed consent to participate in this study was provided by the participants’ legal guardian/next of kin.

## Author Contributions

SP performed conceptualization. KA-P, LA, and SP performed formal analysis. SP performed funding acquisition. KA-P and LA conducted the investigation. The methodology was designed by KA-P, LA, DF, and SP. SP performed project administration and supervision. SP provided resources. KA-P, LA, and SP performed validation. KA-P and SP performed visualization. The writing of the original draft was performed by KA-P and SP. Revisions were performed by SP. All authors contributed to the article and approved the submitted version.

## Funding

This work was supported by NIH grants RO1 AI116282 and R01 AI161014 to SP and seed funds to SP from Baylor College of Medicine and The Scripps Research Institute.

## Conflict of Interest

The authors declare that the research was conducted in the absence of any commercial or financial relationships that could be construed as a potential conflict of interest.

## Publisher’s Note

All claims expressed in this article are solely those of the authors and do not necessarily represent those of their affiliated organizations, or those of the publisher, the editors and the reviewers. Any product that may be evaluated in this article, or claim that may be made by its manufacturer, is not guaranteed or endorsed by the publisher.

## Nomenclature

BMT, bone marrow transplantation; Eomes, Eomesodermin; GVHD, graft-versus-host disease; HCMV, human cytomegalovirus; HSCT, hematopoietic stem cell transplant; NK, Natural killer cell; PBMCs, peripheral blood mononuclear cells; T-bet, T-box transcription factor.
