# Supplementary material for: CXCR6+ and NKG2C+ Natural Killer Cells Are Distinct With Unique Phenotypic and Functional Attributes Following Bone Marrow Transplantation
Source: Front Immunol. 2022 Jun 29;13:886835. doi: 10.3389/fimmu.2022.886835 (PMC9277058; doi:10.3389/fimmu.2022.886835)
Supplement: Supplementary file 1 [file DataSheet_1.pdf]

Supplementary Materials

Supplemental Table 1

**Supplementary Table 1.** Monoclonal antibodies used for multiparameter flow cytometry analysis			
**Marker**	**Fluorophore**	**Clone**	**Company**
CD45	Brilliant Violet 605	HI30	BioLegend
CD3	Brilliant Violet 785	OKT3	BioLegend
CD56	Brilliant Violet 421	HCD56	BioLegend
CXCR6	Alexa Fluor 647	K041E5	BioLegend
CD27	APC-Cy7	O323	BioLegend
Perforin	Alexa Fluor 488	dG9	BioLegend
IFN-gamma	Alexa Fluor 700	4S.B3	BioLegend
TNF alpha	PE-Dazzle 594	Mab11	BioLegend
Ki-67	PE-Cy7	Ki-67	BioLegend
KIR2DL2/DL3	PerCP-Cy5.5	DX27	BioLegend
CD69	PE-Cy5	FN50	BioLegend
NKG2D	Brilliant Violet 510	1D11	BioLegend
TIM-3	PE-Cy7	F38-2E2	BioLegend
T-bet	Brilliant Violet 711	4B10	BioLegend
CD16	APC-Cy7	3G8	BioLegend
CD62L	PE-Cy5	DREG-56	BioLegend
CD57	Alexa Fluor 700	HCD57	Novus
EOMES	PerCP-eFluor 710	WD1928	ThermoFisher Scientific
Granzyme B	Brilliant Violet 510	GB11	BD Biosciences
CD94	Brilliant Violet 650	HP-3D9	BD Biosciences
CD49e	Brilliant Violet 650	IIA1	BD Biosciences
TRAIL	Brilliant Violet 711	RIK-2	BD Biosciences
NKG2A	Alexa Fluor 488	131411	R&D Systems
NKG2C	PE	134591	R&D Systems

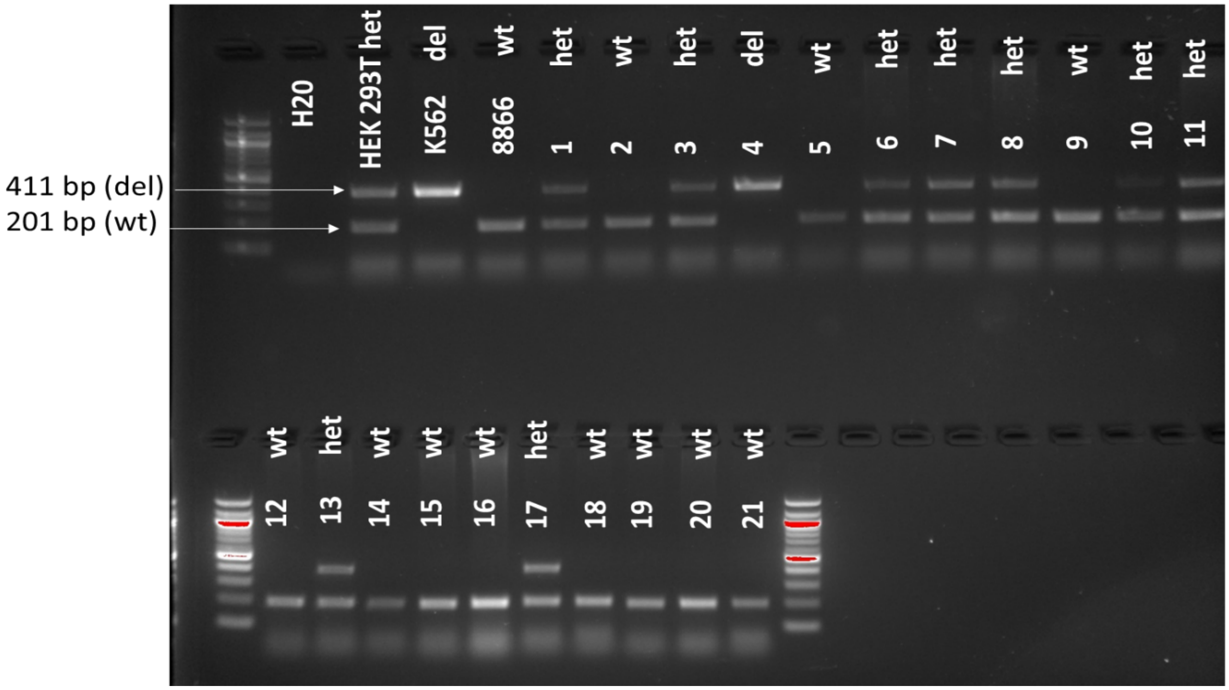

Supplementary Figure 1. KLRG2 copy number variation of HSCT patients. Copy number variation of the Killer Cell Lectin Like Receptor C2 (KLRC2) gene, encoding the NKG2C activating receptor, was assessed using Polymerase Chain Reaction. DNA from the following cell lines were included as controls: heterozygous HEK293T, homozygous null K562, and wild type 8866. KLRG2 deletion mutants generate a 411-base pair (bp) product, 201 bp products indicate wild-type KLRG2 gene products, and if both products are present, then samples are considered heterozygous for KLRG2.

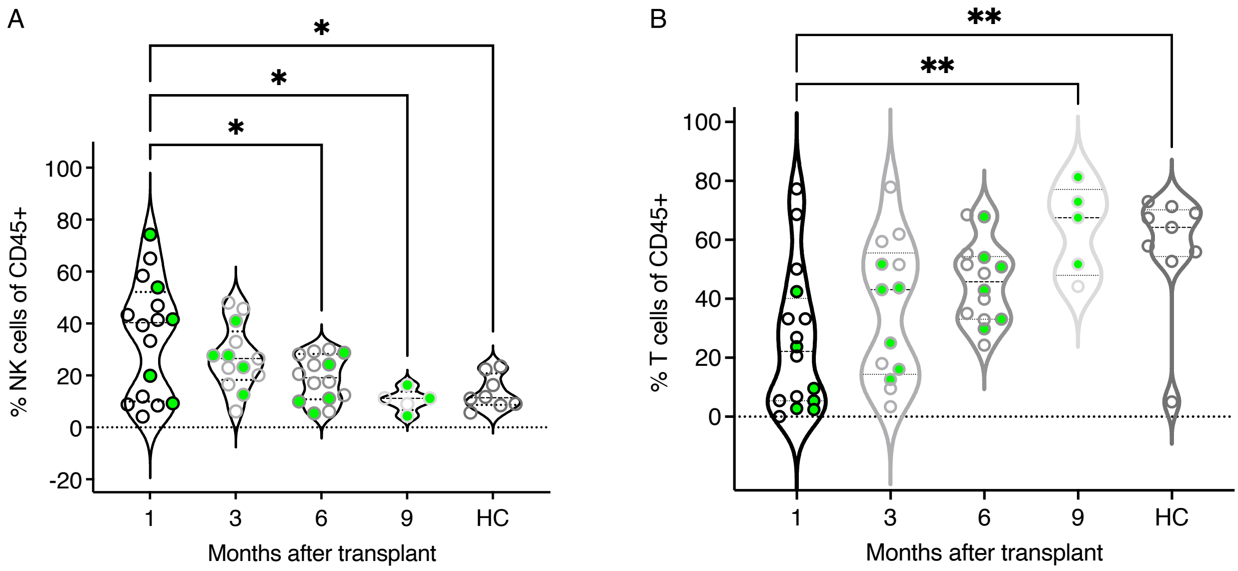

Supplementary Figure 2. NK cell and T cell reconstitutions post-transplant. Percent of NK cells (A) or T cells (B) as a percent of $CD45^+$ cells at indicated time points post-HSCT as determined by flow cytometry. Data shown are mean ± standard deviation and analyzed by one-way ANOVA with multiple comparisons. Each data point represents a single transplant recipient or healthy adult control donor (HC). Data points with a neon-green fill color represent patients that were positive for CMV up to and at the time of analysis as determined by quantitative PCR.

CXCR6^{+} and NKG2C^{+} NK cells are distinct subsets

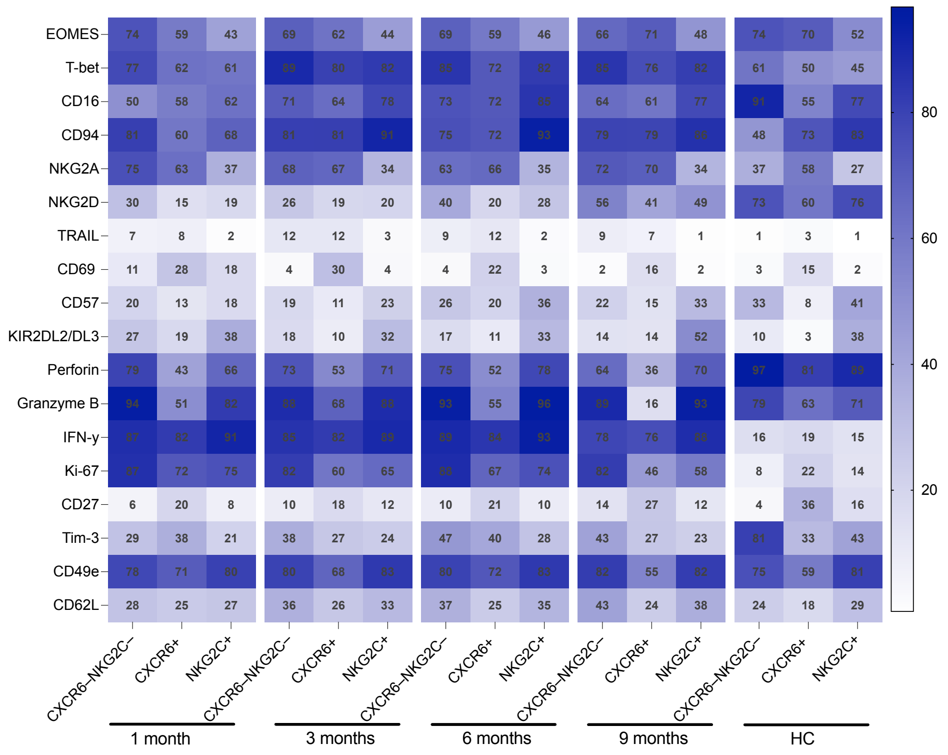

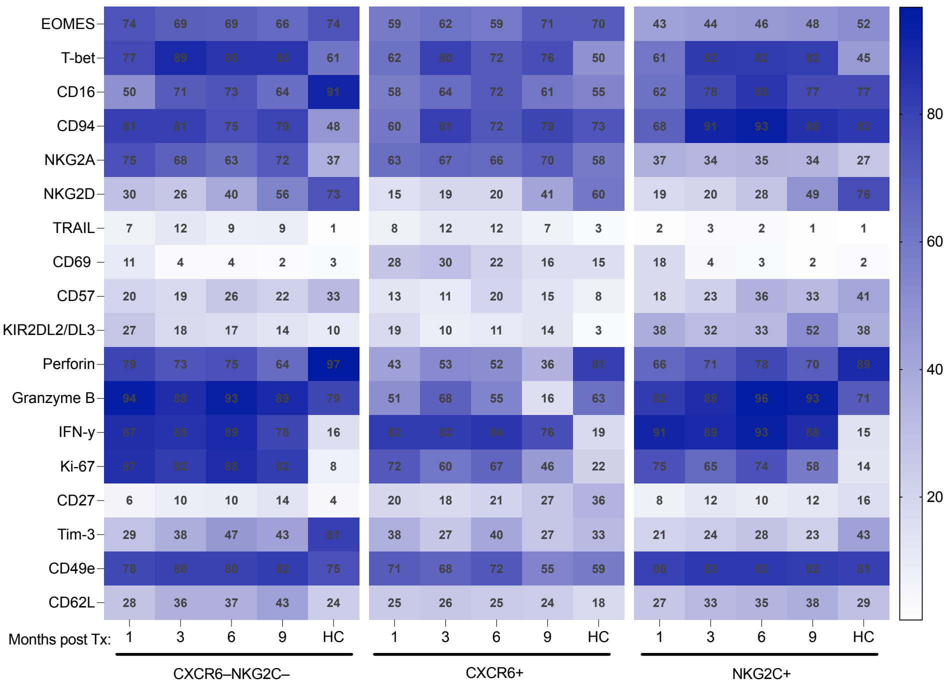

Supplemental Figure 3. Heatmaps of the mean expression frequency of indicated markers in CXCR6^{-}NKG2C^{-} NK cells (DN), CXCR6^{+} NK cell (CXCR6), and NKG2C^{+} NK cell (NKG2C) subpopulations and heathy adult control donors (HC) at the indicated time points after transplant.
